# Development and validation of risk prediction model for refeeding syndrome in neurocritical patients

**DOI:** 10.3389/fnut.2023.1083483

**Published:** 2023-02-15

**Authors:** Wei Zhang, Sheng-Xiang Zhang, Shu-Fan Chen, Tao Yu, Yun Tang

**Affiliations:** ^1^Department of Neurosurgery Intensive Care Unit, The First Affiliated Hospital of Wannan Medical College, Wuhu, China; ^2^Department of Nursing, Soochow University, Suzhou, China

**Keywords:** refeeding syndrome, hypophosphatemia, neurocritical, risk factors, prediction model

## Abstract

**Background:**

The incidence of refeeding syndrome (RFS) in critically ill patients is high, which is detrimental to their prognoses. However, the current status and risk factors for the occurrence of RFS in neurocritical patients remain unclear. Elucidating these aspects may provide a theoretical basis for screening populations at high risk of RFS.

**Methods:**

A total of 357 patients from January 2021 to May 2022 in a neurosurgery ICU of a tertiary hospital in China were included using convenience sampling. Patients were divided into RFS and non-RFS groups, based on the occurrence of refeeding-associated hypophosphatemia. Risk factors for RFS were determined using univariate and logistic regression analyses, and a risk prediction model for RFS in neurocritical patients was developed. The Hosmer-Lemeshow test was used to determine the goodness of fit of the model, and the receiver operator characteristic curve was used to examine its discriminant validity.

**Results:**

The incidence of RFS in neurocritical patients receiving enteral nutrition was 28.57%. Logistic regression analyses showed that history of alcoholism, fasting hours, Acute Physiology and Chronic Health Evaluation II (APACHE II) scores, Sequential Organ Failure Assessment (SOFA) scores, low serum albumin, and low baseline serum potassium were risk factors of RFS in neurocritical patients (*p* < 0.05). The Hosmer-Lemeshow test showed *p* = 0.616, and the area under the ROC curve was 0.791 (95% confidence interval: 0.745–0.832). The optimal critical value was 0.299, the sensitivity was 74.4%, the specificity was 77.7%, and the Youden index was 0.492.

**Conclusion:**

The incidence of RFS in neurocritical patients was high, and the risk factors were diverse. The risk prediction model in this study had good predictive effects and clinical utility, which may provide a reference for assessing and screening for RFS risk in neurocritical patients.

## 1. Introduction

Neurocritical patients require nutritional support due to insufficient oral intake caused by altered states of consciousness and dysphagia (1). Enteral nutrition is the preferred nutritional support method for these patients, which is superior to parenteral nutrition (PN) in maintaining intestinal function and regulating the balance of intestinal flora. In the absence of contraindications, enteral nutrition (EN) should be initiated as soon as possible (within 24–48 h) (2). After a long period of starvation or malnutrition, patients are prone to acute metabolic disorders when they re-intake nutrients through oral, enteral, or parenteral routes, namely refeeding syndrome (RFS), which is potentially fatal (3). While, EN to be more associated with the incidence of RFS than PN (4). RFS usually occurs within 72 h of refeeding in starving or malnourished patients, with a high incidence rates in critically ill patients, and is associated with multisystem damage, and poor prognoses (5, 6). Refeeding a patient after a prolonged period of starvation results in a shift in body processes from catabolism to anabolism. This causes the body to consume excessive amounts of vitamins and electrolytes, as well as develop abnormal glucose and fat metabolisms. Following the initiation of nutritional support, the patient’s blood glucose will rise and stimulate the secretion of insulin, which then increases the cellular uptake of serum phosphate, potassium ions, magnesium ions, vitamins, and trace elements. This results in electrolyte disturbances, such as hypophosphatemia, and multisystem clinical symptoms (7). Additionally, RFS causes deficiencies in thiamine and other trace elements (8, 9), which may cause further multisystem damage. This damage may manifest in conditions such as delirium, heart failure, arrhythmia, respiratory failure, renal failure, abdominal distension, constipation, nausea, vomiting, immunosuppression, and, ultimately, increased mortality (10, 11).

Critically ill patients are more likely to experience RFS due to high rates of stress and metabolism. The incidence of RFS in critically ill patients receiving nutritional support is as high as 36.8 to 59% (12, 13), while the incidence of RFS in neurocritical patients has been reported to be 17.1% in a retrospective cohort study (14). Due to multiorgan damage, RFS may lead to adverse consequences, such as ventilator dependence, increased infection risk, prolonged hospitalization, increased medical burden, and delayed recovery. In addition, studies have confirmed that RFS was an independent risk factor for 6-month mortality rates in neurocritical patients, and that it increased mortality in critically ill patients, with mortality rates ranging from 26 to 100% (5, 8, 15). Therefore, early identification and timely treatment of RFS is particularly important.

However, due to the lack of specific clinical manifestations or a uniformed definition, the level of awareness of RFS by medical staff may vary. The American Society Parenteral Enteral Nutrition (ASPEN) defines RFS as a decrease of any one or combination of serum phosphorus, potassium, or magnesium as well as the presence of manifestations related to thiamine deficiency after recent (hours to days) initiation of nutritional support in long-term malnourished individuals (16). However, lower potassium or phosphate levels may be due to other reasons, such as the use of diuretics or insulin (17). Therefore, a mere decrease in serum potassium does not necessarily indicate the occurrence of RFS. As hypophosphatemia may be a characteristic feature of RFS, many studies characterize RFS as refeeding hypophosphatemia (RH). However, the definition of RH was also not uniform, with phosphorous levels ranging from 0.32 mmol/L to 0.97 mmol/L (5). In a study of all hospitalized patients, Friedli et al. (5) found that the presence of serum phosphorus levels of <0.6 mmol/L, a 30% decrease of serum phosphorus from baseline, or lower than normal potassium or magnesium levels within 72 h of starting nutritional support indicated imminent RFS. Doig et al. (18) further defined RFS in critically ill patients as new-onset hypophosphatemia within 72 h of starting nutritional support (i.e., serum phosphorus <0.65 mmol/L and a decrease from baseline of >0.16 mmol/L). Nevertheless, overt RFS should be considered when clinical symptoms are present.

Currently, preventive measures for RFS mainly include identification of high risk groups, restriction of energy intake, and correction of pre-feeding electrolyte disturbances (19). Many criteria exist for the identification of high risk groups of RFS, with the most widely used being the risk factors proposed by NICE (National Institute for Health and Care Excellence) guidelines (20). These guidelines suggest that those who meet one or more of the following criteria are at high risk for RFS: body mass index (BMI) <16 kg/m^2^; weight loss >15% within the last 3–6 months; reduced eating or fasting for >10 days; decreased serum potassium, phosphate, or magnesium ion concentrations prior to refeeding. Additionally, those who meet two or more of the following risk factors may also be at high risk: BMI <18.5 kg/m^2^; body weight loss >10% within the last 3–6 months; reduced eating or fasting >10 days; history of alcohol abuse; overuse of insulin; and history of chemotherapy, antacid, or diuretic use. However, subsequent studies have shown that the specificity and sensitivity of these guidelines are low, and their ability to identify RFS high risk groups may be poor (4, 21, 22). In addition, although some studies have proposed risk factors and developed risk prediction models for RFS in critically ill patients, these same aspects are lacking for RFS in neurocritical patients. This might be because most neurologists and nurses are unfamiliar with the concept of RFS, risk factors, and associated symptoms, leading to inadequate attention to RFS in neurocritical patients. Therefore, this study aimed to analyze the risk factors of and develop a prediction model for RFS in neurocritical patients. Additionally, we aimed to verify the goodness of fit and discriminative validity of the model in order to provide assessment methods and tools for medical staff in the early identification, diagnosis, and treatment of RFS in neurocritical patients.

## 2. Materials and methods

### 2.1. Study design

This was a retrospective study performed in an 18-bed neurosurgery ICU (NSICU) of a tertiary general hospital in China. Due to the particularity of neurocritical patients and the properties of the observational study, patient informed consent was waived. In this study, we did not conduct additional interventions other than the necessary assessments. Therefore, data collection was not burdensome to patients, and data were collected and analyzed anonymously.

### 2.2. Enrollment

We selected adult patients admitted to the NSICU from January 2021 to May 2022 using convenience sampling. The inclusion and exclusion criteria for this study are as follows. Inclusion criteria: (1) diagnosed with cerebral hemorrhage, intracranial aneurysm, subarachnoid hemorrhage, or acute craniocerebral injury by cranial computed tomography (CT) or magnetic resonance imaging (MRI); (2) received EN for >72 h during admission to NSICU; (3) biochemical test results, including serum phosphorus, before and after EN (within 72 h). Exclusion criteria: (1) incomplete data; (2) aged >80 years or <18 years; (3) serum phosphorus <0.65 mmol/L before admission to NSICU; (4) end-stage malignant tumor; (5) acute respiratory alkalosis, metabolic alkalosis, or diabetic ketoacidosis; (6) presence of other risk factors for hypophosphatemia, such as continuous hemodialysis, hyperphosphatemia treatment, and parathyroidectomy within the last 3 months.

### 2.3. Definition of refeeding syndrome

Participants of this study were neurocritical patients. Therefore, we defined RFS based on the study by Doig et al. (18), which defined RFS as new-onset hypophosphatemia within 72 h of starting nutritional support (i.e., serum phosphorus <0.65 mmol/L and a decrease from baseline of >0.16 mmol/L).

### 2.4. Method of nutritional support

Following the patient’s admission to the NSICU, the physician and the nutrition specialist nurse performed a nutritional assessment based on NRS 2002. Except for those with gastrointestinal bleeding or cerebrospinal fluid leakage due to skull base fractures, almost all patients were given a gastric tube for EN. Doctors selected nutritional formulations and started EN within 24–48 h of a patient’s admission. Short peptide nutritional formulations were used in the early stages of EN and then gradually switched to intact protein formulations based on the patient’s gastrointestinal tolerance. Energy and protein requirements are not precisely estimated; instead, the dose is determined by the physician depending on the patient’s relative pre-albumin or albumin levels. In general, one bottle of nutrient solution (500 Kcal) is delivered on the first day of EN, followed by two bottles of nutrient solution (1,000 Kcal) on the second day. Additionally, protein supplements can be given intravenously to make up for any insufficiencies in EN supply, and the protein dosage is adjusted according to the patient’s condition. The head of the bed was elevated by 30° during EN if the condition permitted and held in that position for 30 min after EN. Furthermore, the nurse regularly monitored the patient for signs of feeding intolerance; if any such symptoms appeared, including bloating, diarrhea, and vomiting, they were reported to the doctor for symptomatic treatment. The feeding rate could be increased by 10–20 ml/h on the following day when the patient did not experience any adverse effects.

### 2.5. Candidate predictors

By analyzing the relevant literature on RFS, combining it with clinical data, and having expert discussions, we selected risk factors that may affect the occurrence of RFS in neurocritical patients. We identified the following 18 candidate predictors: (1) individual patient factors: age, gender, type of disease, comorbidities (diabetes, hypertension, cardiovascular disease, or cerebrovascular disease), history of insulin, history of alcoholism (drinking three standard glasses and above per day or five standard glasses per time, at least once a week), nutritional risk screening (NRS) 2002 scores, Acute Physiology and Chronic Health Evaluation II (APACHE II) scores, Sequential Organ Failure Assessment (SOFA) scores, and Glasgow Coma Scale (GCS) scores; (2) biochemical indicators: serum albumin and prealbumin concentrations on admission, baseline electrolyte levels (serum potassium, sodium, magnesium, and phosphorus levels); and (3) information regarding nutritional support: fasting hours (the time from NSICU admission up until the start of EN), gastrointestinal decompression, feeding route, calorie intake, and protein intake.

### 2.6. Data collection

General patient information was obtained from electronic medical records. We evaluated and recorded GCS scores at NSICU admission and NRS 2002, APACHE II, and SOFA scores of each patient within 24 h of NSICU admission. Additionally, laboratory data within 24 h of NSICU admission and within 72 h of receiving EN were obtained. Patients had a serological biochemical test on the day of admission and were rechecked on average every 2 days after that. We calculated the total daily caloric and protein intake for each patient during the first 72 h of EN. As the type of nutrient solution changed according to the condition of patients, and patients may have used different types of nutrient solutions during the pre-enteral nutrition periods, we did not collect this particular data. Similarly, weight measurements were difficult to obtain, as neurocritical patients are typically bedridden at the time of admission. Therefore, obtaining accurate BMI measurements was not possible. Data were collected by two investigators with mutual verifications of completeness, authenticity, and accuracy of data. Complete data records were maintained by a dedicated individual.

### 2.7. Sample size

According to the sample size calculation criteria based on logistic regression analysis proposed by Gao and Zhang (23), the sample size should be 5–10 times the number of independent variables divided by the incidence of disease, with 10–20% possible invalid cases included. A total of 19 independent variables were included in this study. We selected 50 neurocritical patients for a small sample pre-survey and found that the incidence of RFS was 30%. Therefore, considering 10% of invalid cases, the minimum sample size required for this study was calculated to be 330 cases.

### 2.8. Statistical analysis

Patients were divided into RFS and non-RFS groups based on the diagnostic criteria. Data were analyzed using SPSS Statistics version 22.0 software (IBM, Armonk, New York, United States). The nomogram, calibration curve, and decision curve analysis (DCA) were plotted using R 4.2.1 software (Delaware Public Benefit Corporation, 250 Northern Ave, Boston). Normally distributed, continuous variables were represented as mean ± standard deviation (SD), while results for non-normally distributed, continuous variables were presented as median [interquartile range (IQR)]. Differences between two groups were analyzed using independent *t*-test or Mann–Whitney U tests. Categorical variables were presented using frequencies and percentages, and differences in proportions were analyzed using Chi-Square tests. Univariate statistical analysis was used to compare the data of the two groups of patients, and variables with *p* < 0.05 were included in the binary logistic regression analysis to analyze the risk factors of RFS in neurocritical patients. These independent risk factors, combined with the partial regression coefficient *β* values and intercepts of each risk factor, were used to develop the risk prediction model of RFS. The goodness of fit and discriminant validity of the model were verified using the Hosmer-Lemeshow (HL) test and receiver operating characteristic (ROC) area under the curve (AUC), respectively.

## 3. Results

### 3.1. Patient characteristics

**Figure 1:**
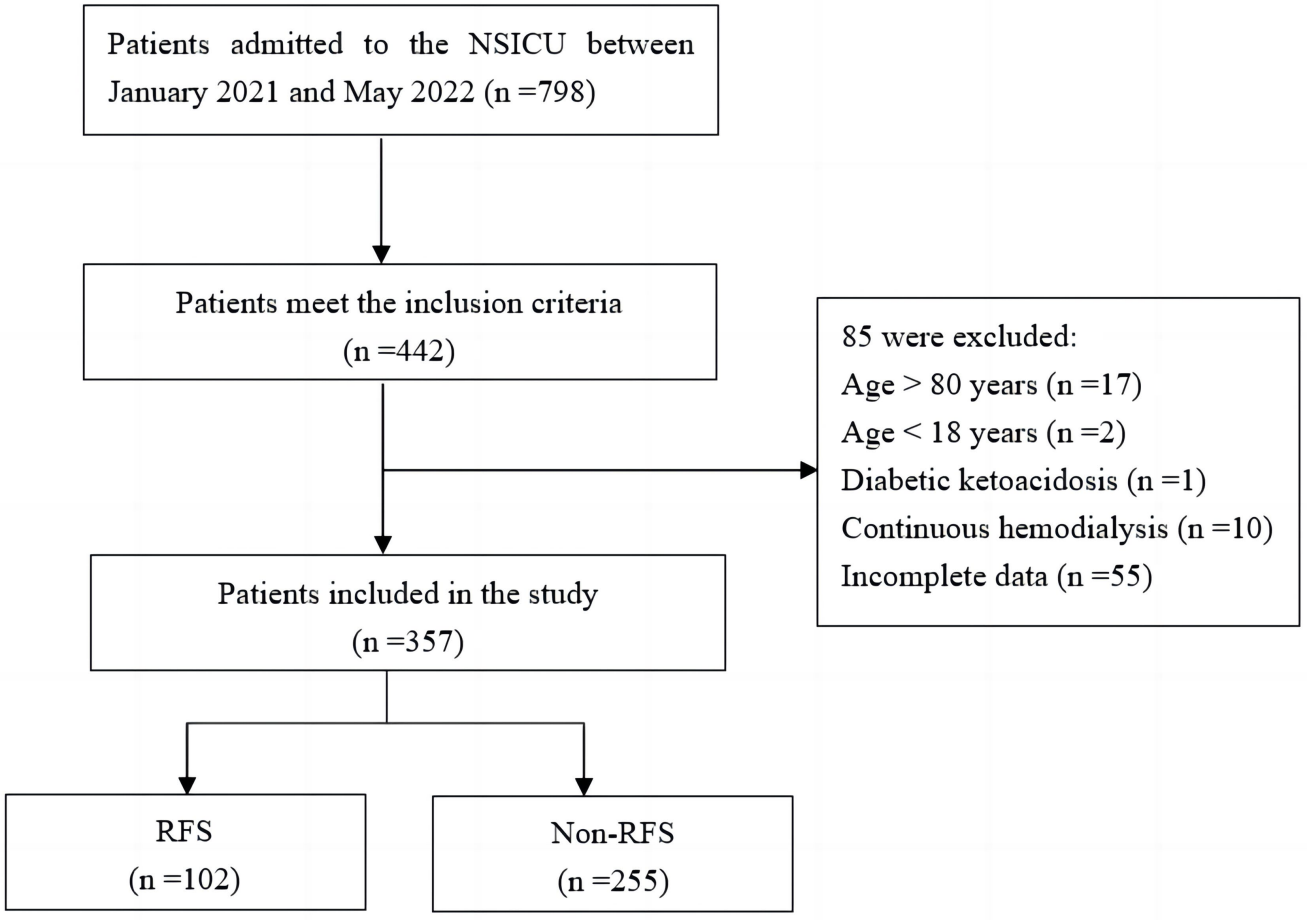
Sampling screening flowchart.

A total of 798 patients were admitted to the NSICU during the study period. Ultimately, 357 patients were included in this study based on the inclusion and exclusion criteria (Figure 1). The RFS and non-RFS groups contained 102 and 255 cases, respectively, and the incidence of RFS among the neurocritical patients receiving EN was 28.57%. Among the patients included in this study, 216 (60.5%) were male and 141 (39.5%) were female. Ages ranged from 21 to 80 (59.61 ± 12.72) years. Of the patients, 195 (54.6%), 142 (39.8%), and 20 (5.6%) had experienced strokes, craniocerebral injuries, and brain tumors, respectively. Moreover, 148 patents (41.5%) had a combined history of hypertension, 37 patients (10.4%) had a history of diabetes, 11 patients (3.1%) had a history of cardiovascular disease, and 23 (6.4%) had a history of cerebrovascular disease. Finally, nasogastric and nasointestinal tube feedings were given in 339 cases (95.0%) and 18 cases (5.0%), respectively.

### 3.2. Univariate analysis of refeeding syndrome

Univariate analysis showed statistically significant differences between the two groups in the variables of diabetes, history of alcoholism, history of insulin, fasting hours, APACHE II scores, SOFA scores, GCS scores, serum albumin, baseline serum potassium, baseline serum phosphorus, and protein intake on the second and third day (*p* < 0.05; Table 1).

**Table 1:** The univariate analysis of the influencing factors of RFS (*n* = 357).

Variables	All Samples (n = 357)	RFS (n = 102)	Non - RFS (n = 255)	*t*/***χ***^2^value	*P* value
Male, *n* (%)	216 (60.5)	64 (62.7)	152 (59.6)	0.300	0.584
Age (years), *n* (%)				1.210	0.546
18-40	30 (8.4)	8 (7.8)	22 (8.6)		
41-65	189 (52.9)	50 (49.0)	139 (54.5)		
>65	138 (38.7)	44 (43.1)	94 (36.9)		
Type of disease, *n* (%)				4.217	0.121
Stroke	195 (54.6)	47 (46.1)	148 (58.0)	4.205	0.040
Craniocerebral injury	142 (39.8)	48 (47.1)	94 (36.9)	3.162	0.075
Brain tumor	20 (5.6)	7 (6.9)	13 (5.1)	0.429	0.512
Comorbidity, *n* (%)					
Hypertension	148 (41.5)	38 (37.3)	110 (43.1)	1.039	0.308
Diabetes	37 (10.4)	16 (15.7)	21 (8.2)	4.354	0.037
Cardiovascular diseases	11 (3.1)	3 (2.9)	8 (3.1)	0.009	0.923
Cerebrovascular diseases	23 (6.4)	5 (4.9)	18 (7.1)	0.562	0.453
History of alcoholism, *n* (%)	37 (10.4)	18 (17.6)	19 (7.5)	8.153	0.004
History of insulin, *n* (%)	20 (5.6)	11 (10.8)	9 (3.5)	7.251	0.007
Gastrointestinal decompression, *n* (%)	64 (17.9)	23 (22.5)	41 (16.1)	2.073	0.150
Fasting hours (h), *n* (%)				8.923	0.012
<24	79 (22.1)	16 (15.7)	63 (24.7)		
24-48	206 (57.7)	56 (54.9)	150 (58.8)		0.243
>48	72 (20.2)	30 (29.4)	42 (16.5)		0.004
Feeding route, *n* (%)				0.006	0.939
Nasogastric tube	339 (95.0)	97 (95.1)	242 (94.9)		
Nasointestinal tube	18 (5.0)	5 (4.9)	13 (5.1)		
Baseline assessment (score)					
NRS 2002	6.57±0.90	6.65±0.82	6.54±0.93	1.039	0.110
APACHE II	14.79±5.24	17.05±4.94	13.89±5.10	5.337	0.000
SOFA	5.89±2.10	7.16±2.32	5.38±1.78	7.771	0.000
GCS	8.31±3.46	7.55±3.45	8.61±3.42	-2.635	0.009
Baseline biochemical indicators					
Serum albumin (g/L)	36.28±4.86	35.28±5.60	36.68±4.48	-2.477	0.014
Prealbumin (g/L)	19.97±6.01	19.91±5.98	20.00±6.03	-0.133	0.894
Kreatinine (umol/L)	67.14±51.67	66.62±53.35	67.35±51.09	-0.122	0.903
Baseline electrolyte (mmol/L)					
Serum potassium	3.81±0.48	3.71±0.51	3.85±0.47	-2.458	0.014
Serum sodium	143.93±5.25	143.77±4.73	144.00±5.45	-0.371	0.711
Serum magnesium	0.93±0.19	0.90±0.19	0.94±0.19	-1.914	0.056
Serum phosphorus	0.88±0.25	0.82±0.26	0.91±0.25	-3.294	0.001
Calorie intake within the first 3 days of EN (Kcal)					
Day 1	680.67±246.30	720.59±249.49	664.71±243.68	1.944	0.053
Day 2	925.77±196.76	946.08±155.86	917.65±210.65	1.234	0.218
Day 3	934.45±191.29	942.16±171.45	931.37±198.91	0.481	0.631
Protein intake within the first 3 days of EN (g)					
Day 1	43.53±27.02	46.37±16.52	42.39±30.17	1.258	0.209
Day 2	50.95±14.41	53.43±13.97	49.96±14.49	2.066	0.040
Day 3	50.50±13.91	53.63±15.60	49.25±13.01	2.706	0.007

### 3.3. Logistic regression analysis of refeeding syndrome

**Figure 2:**
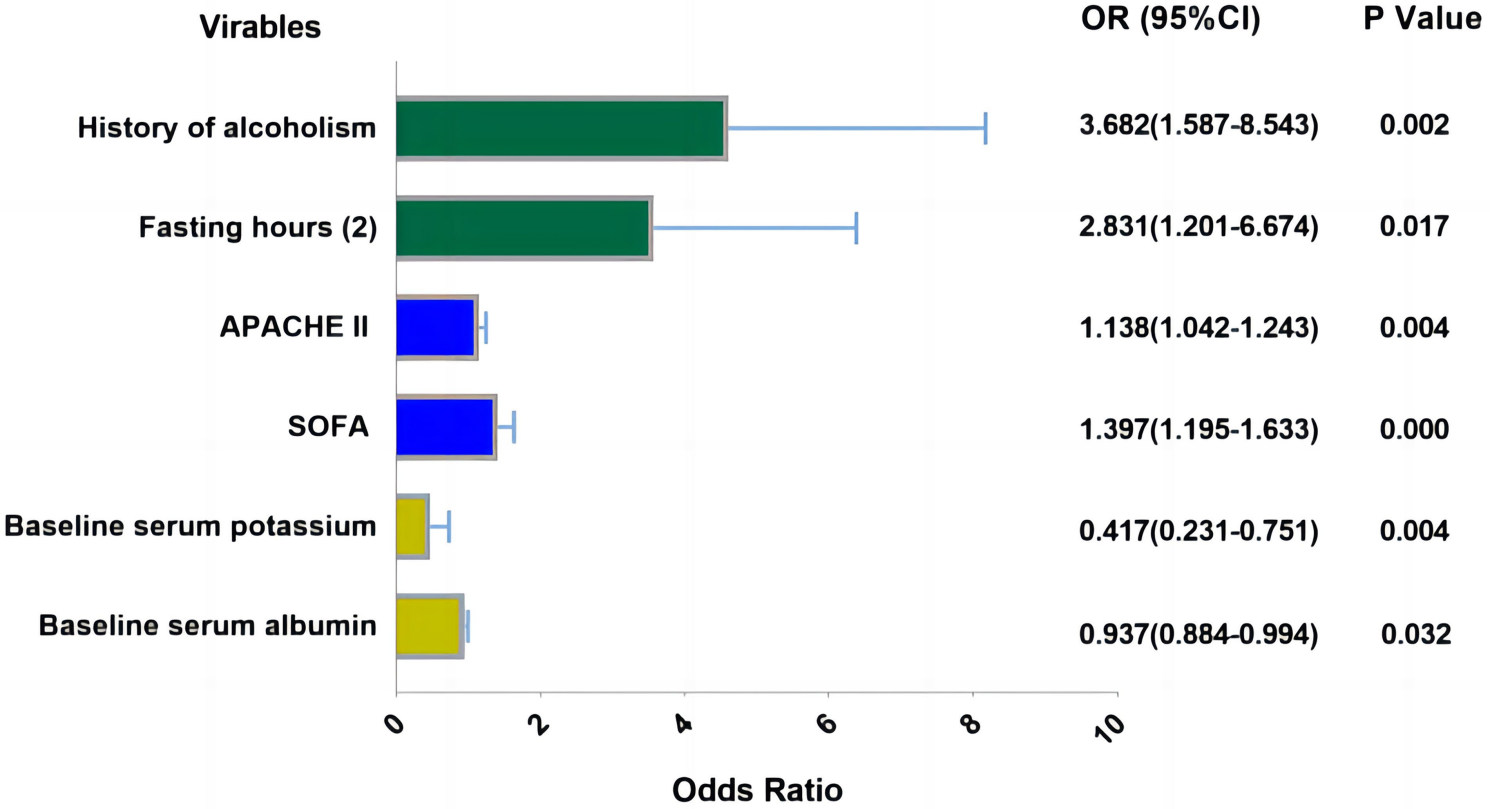
Visualization results of logistic regression analysis. Fasting hours: (1): 24–48 h, Fasting hours: (2) >48 h (Reference category: <24 h), APACHE II, SOFA-score, serum potassium and albumin were analyzed as the continuous variable which were all obtained at admission of patient.

Logistic regression analyses were conducted with the incidence of RFS as the dependent variable and the variables with significant differences in univariate analyses as the independent variables. The assignments of variables are shown in Supplementary material S1. Logistic regression analyses showed that six factors were associated with RFS, including history of alcoholism, fasting hours, APACHE II scores, SOFA scores, serum albumin, and baseline serum potassium. The visualization results of the logistic regression analyses are shown in Figure 2.

### 3.4. Development of model

The partial regression coefficients of the RFS independent predictors determined based on binary logistic regression analyses were used to develop the model. The fitted regression equation of the RFS risk prediction model for neurocritical patients was as follows: *p* = 1 / [1+ exp. (−0.901 + 1.303 × history of alcoholism +0.130 × APACHE II + 0.334 × SOFA +1.041 × fasting hours - 0.876 × baseline serum potassium-0.065 × serum albumin)]. The nomogram model of the risk prediction of RFS is shown in Supplementary material S2.

### 3.5. Validation of model

#### 3.5.1. Goodness of fit test

The goodness of fit of the model was tested using the HL test. The results of this model show that *p* = 0.616 (>0.05), indicating that the predictive ability of the RFS risk prediction model was more consistent with the actual incidence and had a better fit. The calibration curve of the risk prediction model of RFS is shown Supplementary material S3.

### 3.6. Discriminant validity test

**Figure 3:**
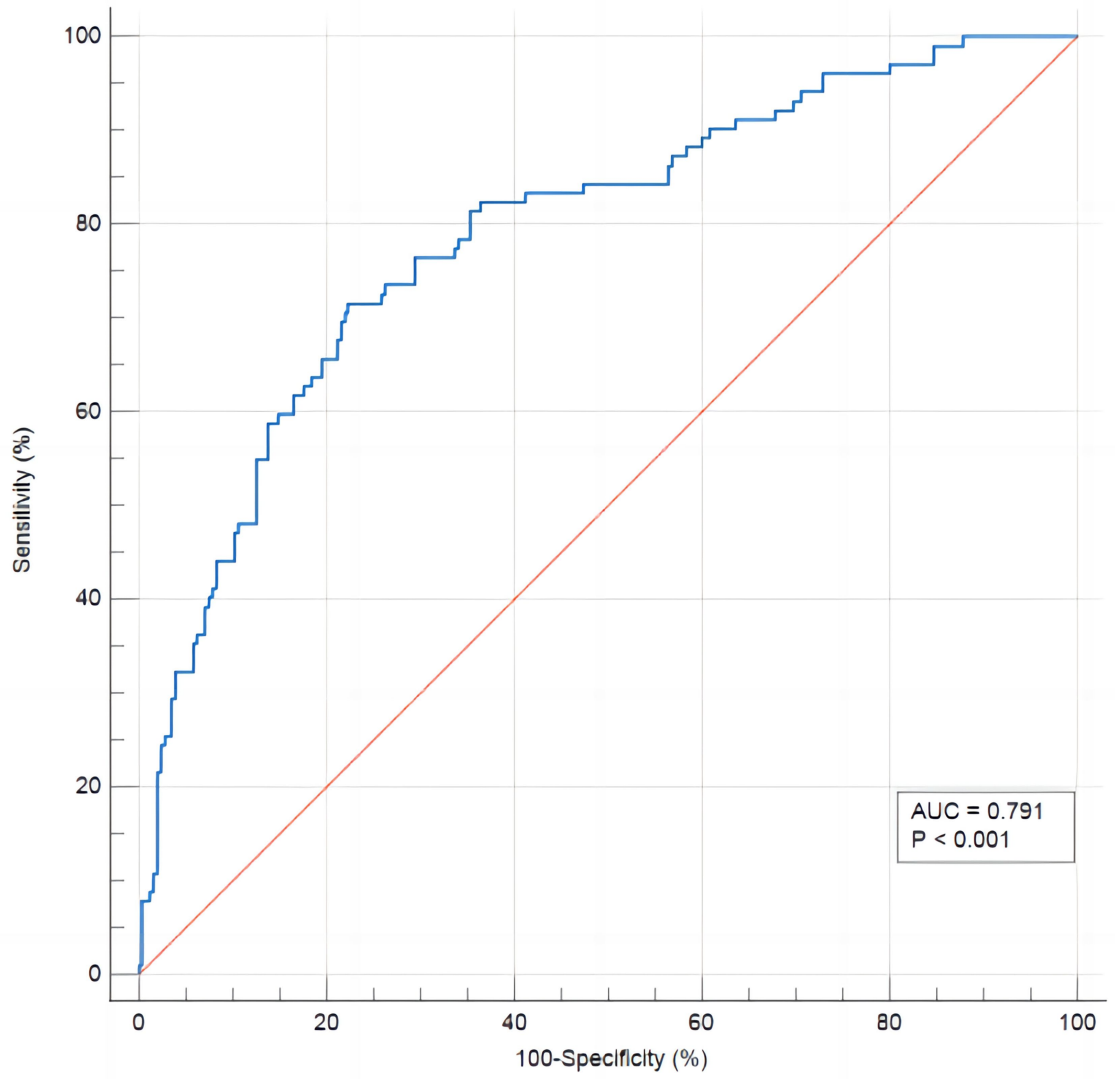
The ROC curve of the risk prediction model of RFS.

**Figure 4:**
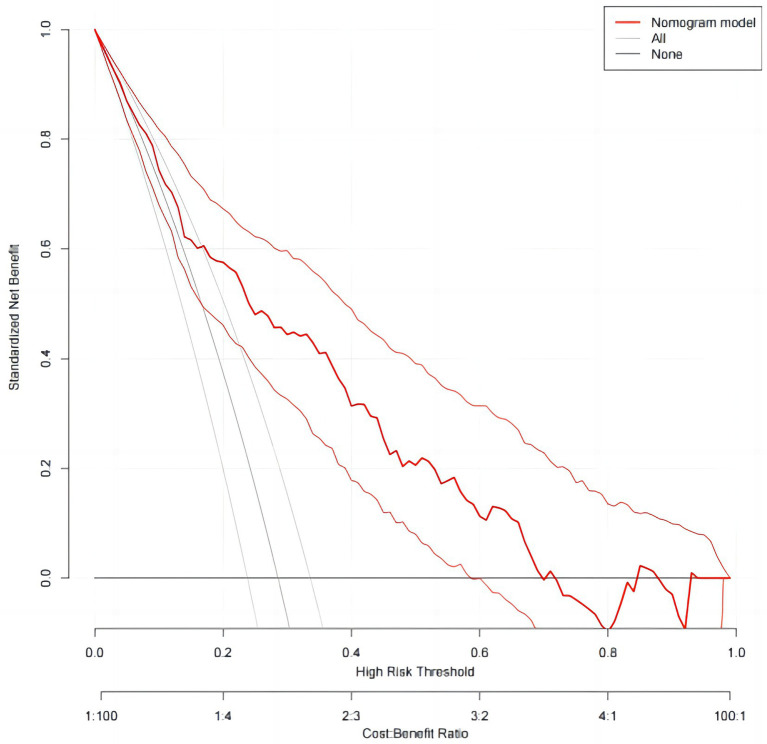
The DCA of the risk prediction model of RFS.

In order to evaluate the discriminant validity of the model, the AUC was calculated by plotting the ROC curve. The results of this model show that AUC was 0.791 (95% confidence interval: 0.745–0.832; *p* < 0.001), suggesting that the model was capable of discriminating whether RFS occurred. When the optimal risk cut-off value was 0.299, the sensitivity and specificity of the model were 71.8 and 77.7%, respectively, and the Youden index was 0.492 (Figure 3). The decision curve analysis of the risk prediction model of RFS is shown in Figure 4.

## 4. Discussion

Critically ill patients receiving nutritional support are susceptible to RFS characterized by hypophosphatemia. This study showed that the incidence of RFS in neurocritical patients was 28.57%, which was mostly consistent with previous studies in ICU patients (12, 24, 25). However, Xiong et al. (14) reported that the incidence of RFS in neurocritical patients was 17.1%, lower than the results of this study. This could be because patients in this study were more severely ill due to higher APACHE II, SOFA, and GCS scores, while our study also found a significant association between the severity of illness and RFS. The incidence of RFS in neurocritical patients in existing studies varies due to the lack of a diagnostic gold standard, the uncertainty of the optimal diagnostic concentration of hypophosphatemia, and differences in pre-feeding prophylactic interventions (24). However, all of these factors, to some extent, indicate a higher risk of RFS in neurocritical patients. Therefore, more focus on RFS by medical personnel may be needed. In addition to monitoring vital signs, serum phosphate concentrations and various routine laboratory indicators, medical personnel should also pay attention to the patient’s kidney function and metabolic acid–base balance. Impaired renal clearance of phosphorus may prevent electrolyte depletions associated with the RFS. Also, phosphorus is subject to acid–base shifts. Patients with (metabolic) acidosis and an absolute deficit of phosphorus may present with normal phosphorus levels at baseline, but when acidosis restores, hypophosphatemia may occur as a result to acid–base shift. However, in any instance, we should adhere to the current definition of RFS, which states that hypophosphatemia from any cause should not be regarded as RFS if the patient is not receiving EN. Further research is definitely needed to determine whether kidney function and acidosis can lead to RFS.

Early identification of patients at risk for RFS is particularly important as RFS has no specific clinical symptoms and can be judged only by phosphate levels. NICE guidelines (20) identified BMI; unintentional weight loss; hunger; history of alcohol abuse; and pre-feeding serum phosphorus, potassium, and magnesium levels as risk factors for RFS. However, Goyale et al. (21) showed that the predictive power of the NICE guidelines was poor. In addition, some studies has been suggested that pre-feeding serum albumin levels, age, chemotherapy, and the use of acid suppressants, diuretics, and insulin are associated with the risk of RFS (26–28). Friedli et al. (26) developed a new risk stratification scheme based on a modification of the NICE guidelines; however, this study found that the sensitivity for predicting RFS in patients with severe stroke remained low. This may be related to there being no uniform diagnostic criteria and many influencing factors in the development of RFS. Besides, this risk stratification did not include all risk factors, such as severity of illness scores, which may explain the poor predictive power. In this study, 18 factors that may be associated with the occurrence of RFS in neurocritical patients were selected, based on NICE guidelines, related studies, expert opinions, and current patient conditions. Based on univariate and binary logistic regression analyses, six independent risk factors for RFS in neurocritical patients were ultimately obtained, including a history of alcoholism, fasting hours, APACHE II scores, SOFA scores, serum albumin, and baseline serum potassium.

Although ASPEN clinical practice guidelines suggest that early EN facilitates recovery from disease in critically ill patients (29), studies have found that the time from admission to initiation of EN in patients remains long (30). Due to prolonged fasting in patients, the intake of various nutrients is low, and high catabolic states deplete the patient of electrolytes and minerals. Consequently, when refeeding, these patients are more prone to electrolyte disturbances and sodium and water retention as the electrolytes are further displaced (27). In addition, the lack of food and gastrin stimulation of the intestinal mucosa causes a decrease in the secretion of gastric acid, bile, and other digestive juices, resulting in weakened bactericidal capacities of the intestines, as well as a proliferation and displacement of intestinal toxins, causing an intestinal or systemic inflammatory response that affects the digestive and absorption functions of the intestine (31). These factors are ultimately detrimental to the implementation of EN. Therefore, following guidelines regarding early EN in critically ill patients and avoiding long periods of fasting is essential.

This study showed that both APACHE II and SOFA scores were risk factors for RFS in neurocritical patients, which is concordant with the recent study (14, 32). APACHE II scores are an important tool for assessing the severity of disease in patients, and SOFA scores reflect the organ function status and prognosis of patients. In this study, we observed that high APACHE II and SOFA scores were associated with increased odds of RFS. This finding may be due to the increase in nutritional requirements, impairment of food intake, and increased stress-metabolism resulting from disease (33). Due to the traumas, surgeries, and critical conditions associated with neurocritical patients, the body may be in a state of intense stress and high catabolism, requiring large amounts of energy and electrolytes. In these cases, after the implementation of nutritional support, glucose concentrations begin to rise, causing an increase in insulin secretion and redistribution of electrolytes inside and outside the cell through sodium-potassium-ATPase isotransport proteins. Additionally, large amounts of K^+^, Mg^2+^, and phosphate enter the cell, while Na^+^ is transported outside of the cell, resulting in electrolyte disorders, primarily hypophosphatemia (24).

The NICE guidelines on nutrition support for adults (20) suggest that nutritional risk screening can effectively predict RFS. Rasmussen et al. (22) also showed that NRS-2002 scores have a high predictive value for RFS. However, this study was not able to confirm whether initial nutritional risk statuses of neurocritical patients were related to RFS. This finding may be due to the specificity of diseases in neurocritical patients. That is, both stroke and craniocerebral injury are factors that increase the nutritional risk of patients, and the differences in nutritional risk between patients are low. Therefore, more relevant studies are needed to further validate the findings. In addition, the results of this study showed that baseline albumin levels were associated with RFS in neurocritical patients, which was consistent with the results of previous studies (14, 27). Albumin level is an objective indicator of the nutritional status of patients, where low albumin levels indicate malnourishment and high nutritional needs in patients require nutritional support in patients (34). Marik et al. (35) also found that the only risk factor that could predict the development of hypophosphatemia in critically ill patients was the serum prealbumin concentration, which further suggests that malnourished patients are more likely to develop RFS. However, most of the current nutritional support is provided through increased caloric and protein intake, which may result in imbalanced, excessive, or rapid supplementation. This may then cause increased insulin secretion and rapid electrolyte depletion and redistribution, which may lead to the occurrence of RFS. As in the results of this study, we observed that patients with RFS received more protein on days 2 and 3 of EN than patients without RFS. This might be because physicians focused solely on the patients’ albumin levels and provided them protein rapidly, which increased the likelihood that RFS would occur. However, a comprehensive assessment should be done based on the patients’ ideal weight and their nutritional status. As a result, the serum phosphorus level should be constantly monitored as needed, and patients at risk should be fed slowly, although protein and electrolyte supplementation is necessary for patients at high risk of RFS.

This study showed that a history of alcoholism is a risk factor for RFS, which may be due to the electrolyte disturbance caused by excessive alcohol consumption. Studies have shown that drinking more than 9 standard cups per week (one standard cup is equivalent to 12 g of pure alcohol) was considered as excessive drinking, which will cause varying degrees of damage to the body, among which water and electrolyte disorders are one of the main complications (36). The incidence of hypokalemia is significantly higher in patients with severe alcohol dependence than in others (37). While, our study findings were consistent with related studies (27, 38) that showed that low baseline serum potassium was also a risk factor for RFS. However, the association between serum phosphorus and magnesium levels and RFS in neurocritical patients could not be confirmed. Besides, further studies are needed to verify whether drinking history can directly affect serum phosphorus level of patients and the influencing mechanism.

In this study, we developed a model for predicting the risk of RFS in neurocritical patients based on the results of the binary logistic regression analysis. We also verified its goodness of fit, using the Hosmer–Lemeshow test, and its discriminant validity, by plotting ROC curves and calculating AUC. ROC is a tool used to describe the discriminatory accuracy of a diagnostic test or predictive model, and is a combination of the response sensitivity and specificity, which plays an important role in determining the accuracy of a diagnostic test or predictive model (39). The AUC is a visual representation of the accuracy of the prediction model, and is a graph of the interaction between specificity and sensitivity, which ranges from 0.5–1, with values closer to 1 indicating higher discriminatory ability. Generally, AUC values >0.7 indicate that a model has good discriminatory ability (40). The HL test is used to assess the degree of conformity between the observed event rate of the model and the actual rate, where *p* > 0.05 indicates that the model’s predicted events do not differ from the actual events and can reflect the actual situation well (41). In this study, the AUC of the model was 0.791 (95% confidence interval, 0.74–-0.832). Additionally, the HL test showed a *p* = 0.616, indicating that the model had good practical predictive ability. The DCA also showed clinical benefits of the model. The development of the RFS risk prediction model in this study provided a scientific reference for the prevention and treatment of RFS in neurocritical patients, which is conducive to proactive identification, prevention, and timely intervention by healthcare professionals. For example, prompt identification of high-risk groups based on RFS risk factors and providing timely and targeted interventions, including replenishment of electrolytes and phosphates, shortening the fasting time, and starting EN as early as possible. This may help to prevent and reduce the occurrence of RFS in neurocritical patients, ultimately improving their prognosis. Additionally, the clinical relevance of this prediction model is that, with the exception of patients with special comorbidities, such as cirrhotic ascites or shock, who require rapid and large amounts of albumin supplementation, other neurocritical patients should be supplemented with energy and protein based on the patient’s ideal weight and nutritional status during EN. It is recommended that nourishing feeding begin early, gradually transitioning to the patient’s target requirements, to ensure adequate and balanced access to energy and nutrients.

## 5. Limitations

This study had some limitations. First, this study was a retrospective study, and some patients were excluded due to the lack of serum phosphorus test results at admission or within 72 h of EN, resulting in potential selection bias. Second, this study only used one tertiary hospital as the data source to develop the prediction model, and the sample may not be sufficiently representative. The results of this study may need further validation. Third, due to objective factors, such clinical conditions, some possible risk factors were not included in this study, such as BMI. This may have had an impact on the predictive efficacy of the prediction model. Additionally, the clinical outcomes of patients, such as ICU length of stay and mortality were not further explored in this study, which may be a direction for future efforts. Finally, though internal validation of the model was conducted, we did not confirm external validation. Therefore, the applicability of the prediction model may need further exploration.

## 6. Conclusion

The incidence of RFS in neurocritical patients was high, and history of alcoholism, fasting hours, APACHE II scores, SOFA scores, low serum albumin, and low baseline serum potassium were the main risk factors for RFS in neurocritical patients. Therefore, early screening for RFS risk in these patients as well as enhanced assessment of nutritional status may be particularly important to reduce the incidence of RFS. This study developed a predictive model for RFS risk in neurocritical patients, which had good predictive effect and clinical utility. The model may be used as an objective and convenient screening tool in the early identification of high-risk patients, development of appropriate interventions to reduce RFS occurrence, and improvement of patient prognoses.

## Data availability statement

The raw data supporting the conclusions of this article will be made available by the authors, without undue reservation.

## Author contributions

WZ was responsible for the design of the study, data curation, and analysis and completed the draft of the manuscript. S-XZ and S-FC participated in data collection and coordination of the study. YT and TY were responsible for quality control of this study and review of the manuscript. All authors have read and approved the final version of the manuscript.

## Funding

This study was supported by Young and Middleaged Scientific Research Project of Wannan Medical College (No. WKS2021F03).

## Conflict of interest

The authors declare that the research was conducted in the absence of any commercial or financial relationships that could be construed as a potential conflict of interest.

## Publisher’s note

All claims expressed in this article are solely those of the authors and do not necessarily represent those of their affiliated organizations, or those of the publisher, the editors and the reviewers. Any product that may be evaluated in this article, or claim that may be made by its manufacturer, is not guaranteed or endorsed by the publisher.
