# Supplementary material for: Development and validation of risk prediction model for refeeding syndrome in neurocritical patients
Source: Front Nutr. 2023 Feb 15;10:1083483. doi: 10.3389/fnut.2023.1083483 (PMC9975392; doi:10.3389/fnut.2023.1083483)
Supplement: Supplementary file 1 [file Table_1.doc]

The assignments of independent variables

| Variables | Indicators | Assignment Description |
| --- | --- | --- |
| Dependent variable |  | 0 = Non - RFS, 1= RFS |
| Independent variables | Diabetes | 0 = No, 1= Yes |
|  | History of alcoholism | 0 = No, 1= Yes |
|  | History of insulin | 0 = No, 1= Yes |
|  | Fasting hours | 0 = <24 h, 1= 24-48h, 2= >48h |
