# Supplementary figures and images for: Development and validation of risk prediction model for refeeding syndrome in neurocritical patients
Source: Front Nutr. 2023 Feb 15;10:1083483. doi: 10.3389/fnut.2023.1083483 (PMC9975392; doi:10.3389/fnut.2023.1083483)

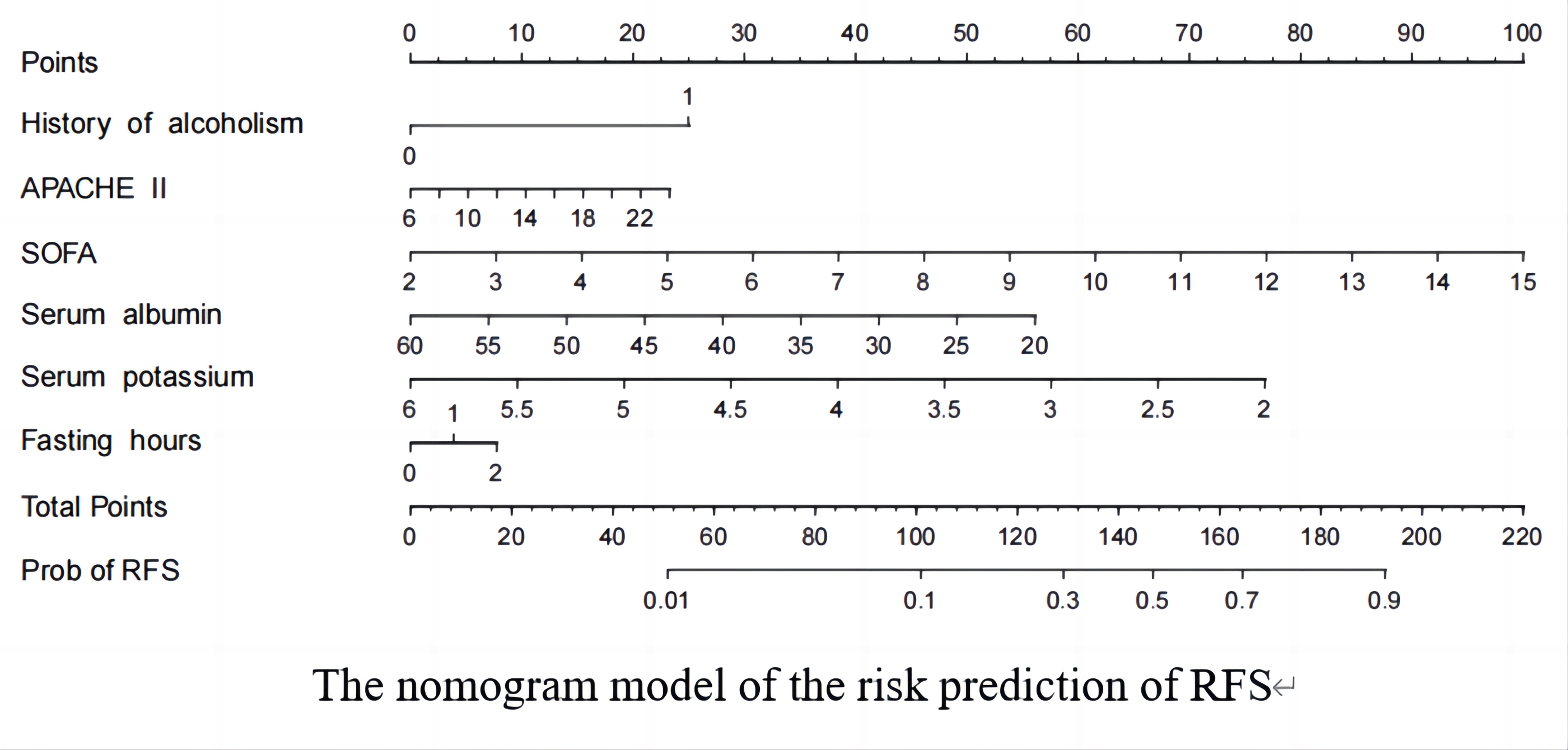

Supplement: Supplementary file 2 [file Image_1.JPEG]

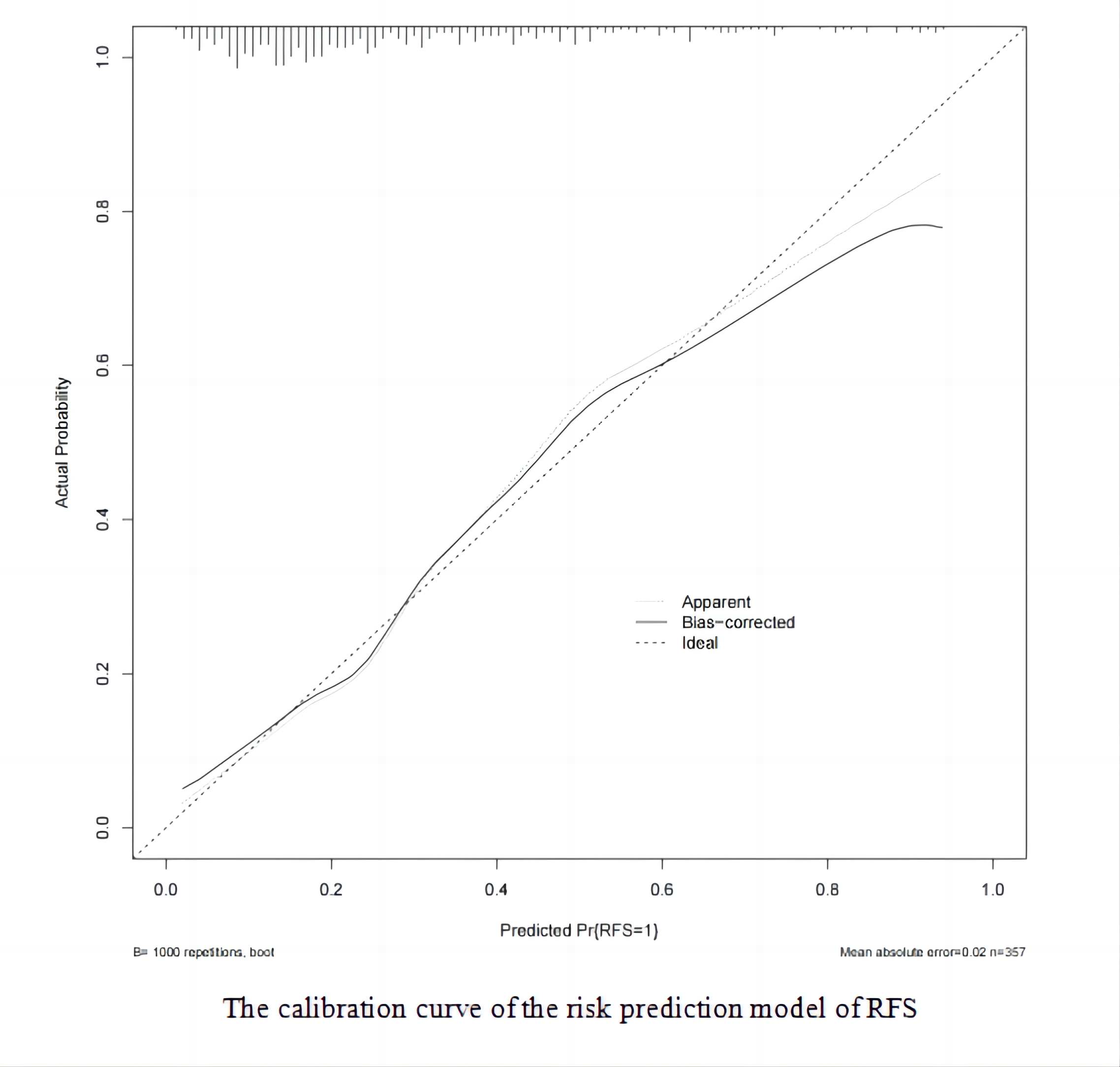

Supplement: Supplementary file 3 [file Image_2.JPEG]
